# Determining effects of nitrate, arginine, and ferrous on antibiotic recalcitrance of clinical strains of *Pseudomonas aeruginosa* in biofilm-inspired alginate encapsulates

**DOI:** 10.1186/s12941-023-00613-y

**Published:** 2023-07-20

**Authors:** Fereshteh Jabalameli, Mohammad Emaneini, Reza Beigverdi, Shahnaz Halimi, Maryam Siroosi

**Affiliations:** 1 Department of Microbiology, School of Medicine, Tehran University of Medical Sciences, Tehran, Iran; 2 Research Center for Antibiotic Stewardship and Antimicrobial Resistance, Tehran University of Medical Sciences, Tehran, Iran

**Keywords:** Antibiotic resistance, Arginine, Biofilm, Ferrous, *Pseudomonas aeruginosa*

## Abstract

**Background:**

Biofilms play a role in recalcitrance and treatability of bacterial infections, but majority of known antibiotic resistance mechanisms are biofilm-independent. Biofilms of *Pseudomonas aeruginosa*, especially in cystic fibrosis patients infected with the alginate producing strains in their lungs, are hard to treat. Changes in growth-related bacterial metabolism in biofilm affect their antibiotic recalcitrance which could be considered for new therapies designed based on these changes. In this study, effects of nitrate, arginine, and ferrous were investigated on antibiotic recalcitrance in alginate-encapsulated *P. aeruginosa* strains isolated from cystic fibrosis patients in the presence of amikacin, tobramycin, and ciprofloxacin. Also, expression of an efflux pump gene, *mexY*, was analyzed in selected strains in the presence of amikacin and ferrous.

**Methods:**

Clinical *P. aeruginosa* strains were isolated from cystic fibrosis patients and minimum inhibitory concentration of amikacin, tobramycin, and ciprofloxacin was determined against all the strains. For each antibiotic, a susceptible and a resistant or an intermediate-resistant strain were selected, encapsulated into alginate beads, and subjected to minimal biofilm eradication concentration (MBEC) test. After determining MBECs, sub-MBEC concentrations (antibiotics at concentrations one level below the determined MBEC) for each antibiotic were selected and used to study the effects of nitrate, arginine, and ferrous on antibiotic recalcitrance of encapsulated strains. Effects of ferrous and amikacin on expression of the efflux pump gene, *mexY*, was studied on amikacin sensitive and intermediate-resistant strains. One-way ANOVA and t test were used as the statistical tests.

**Results:**

According to the results, the supplements had a dose-related effect on decreasing the number of viable cells; maximal effect was noted with ferrous, as ferrous supplementation significantly increased biofilm susceptibility to both ciprofloxacin and amikacin in all strains, and to tobramycin in a resistant strain. Also, treating an amikacin-intermediate strain with amikacin increased the expression of *mexY* gene, which has a role in *P. aeruginosa* antibiotic recalcitrance, while treating the same strain with ferrous and amikacin significantly decreased the expression of *mexY* gene, which was a promising result.

**Conclusions:**

Our results support the possibility of using ferrous and arginine as an adjuvant to enhance the efficacy of conventional antimicrobial therapy of *P. aeruginosa* infections.

**Supplementary Information:**

The online version contains supplementary material available at 10.1186/s12941-023-00613-y.

## Background

*Pseudomonas aeruginosa* is one of the most common Gram-negative opportunistic pathogens causing wound, blood stream, urinary tract, and lung infections mostly in cystic fibrosis patients; moreover, it is recognized for its various virulence factors and resistance against antibiotics [1]. The lowly permeable outer membrane which restricts uptake of antibiotics, antibiotic-inactivating enzymes, and resistance-nodulation-division (RND) family of efflux pumps that specifically pump antibiotics out of the cell are some of the antibiotic resistance mechanisms [2, 3]. It has been shown that four RND efflux pumps in *P. aeruginosa* including MexAB-OprM, MexCD-OprJ, MexEF-OprN, and MexXY-OprM are responsible for expelling different antibiotic families from the cell [3]. Among them, MexAB-OprM is constitutively expressed and recognizes a broad range of antibiotics (fluoroquinolones, tetracycline, chloramphenicol, and β-lactams) as substrates to pump them out of the cell [4], however, other efflux pumps are expressed under specific conditions and act more specifically towards antibiotics. For example, MexXY-OprM is responsible for *P. aeruginosa* resistance to aminoglycoside antibiotics along with aminoglycoside modifying enzymes [3–5]. Chronic, difficult-to-treat *P. aeruginosa* infections which are threads to the public health are related to overexpression of these four efflux pumps and the ability of this bacterium to form biofilms [2, 5]. Biofilm formation is another bacterial strategy to survive antimicrobial substances and host immune responses which is the reason that biofilm associated infections, including *P. aeruginosa* ones represent more than 65% of clinical infections [6, 7]. Forming a biofilm is a complex process involving both environmental factors and regulation of bacterial genes [6].

Various proteins and polysaccharides like alginate are produced to form extracellular matrix of * P. aeruginosa* biofilm [6]. Aggregates of *P. aeruginosa* extracted from patients with biofilm infections contain 0.4–10% alginate which protects bacterial cells from antibiotics [8]. Also, this matrix limits oxygen diffusion into biofilms which makes hypoxic or anoxic microenvironments that restricts bacterial growth and causes antibiotic tolerance [9]. Because *P. aeruginosa* reveals different behaviors regarding biofilm or planktonic lifestyle, various in vitro models have been developed to study the biofilm lifestyle of bacteria**.** Among them, alginate-encapsulated *P. aeruginosa* develops aggregates with similarities to in vivo biofilms [10, 11]. The way that bacteria aggregates into alginate and their resistance to antimicrobial agents are similar to natural biofilms in lung and wound infections [10, 12].

As clearance of *P. aeruginosa* infections are problematic, novel therapeutic approaches including phage therapy and development of vaccines and new antimicrobial agents like antibiotics, nanoparticles, and peptides have been adopted [3]. It is always important to study factors affecting *P. aeruginosa* behavior to develop new therapies for infections caused by this bacterium. For instance, some nutritional factors like succinate, glutamate, and glucose influence the gene expression and induce dispersion in biofilms or fumarate and succinate sensitize cells to the antibiotic tobramycin [13–15]. The presence of electron acceptors like arginine and nitrate affects growth and metabolic activity of biofilms in hypoxic microenvironments which can be helpful to increase susceptibility of cells to antibiotics and eradicate the infections [11]. It has been suggested that the transition from a planktonic (free-swimming) mode to growth as a biofilm occurs as a response to the availability of nutrients. While nutrient-depleted environments enhance biofilm formation, it has been demonstrated that the availability of nutrients in high concentrations represses the formation of biofilms [14]. According to the previous studies, iron in high concentrations limits biofilm formation by *P. aeruginosa*, and suppresses development of a subpopulation with increased antibiotic resistance [16]. Moreover, biofilms grown in the presence of iron show increased susceptibility towards antibiotics [16] and treating biofilms with high doses of iron disrupts the biofilm [17].

The goal of this research was to use a biofilm model to study effects of NO_3_^−^, Fe^2+^, and arginine on antibiotic recalcitrance of clinical *P. aeruginosa* strains. For this purpose, minimum inhibitory concentration (MIC) and minimal biofilm eradication concentration (MBEC) values of ciprofloxacin, tobramycin, and amikacin were determined for clinical *P. aeruginosa* strains. For each antibiotic, one resistant strain (except for amikacin that none of the strains were resistant to this antibiotic and we had to select the intermediate strain) and one sensitive strain which was sensitive to all the three tested antibiotics were randomly selected and the effects of nitrate, arginine, and ferrous on biofilm susceptibility to ciprofloxacin, tobramycin, and amikacin for selected clinical *P. aeruginosa* strains were investigated in alginate beads. Also, *mexY* expression was analyzed in the amikacin-intermediate strain when it was treated with just amikacin and a combination of amikacin and ferrous. To the best of our knowledge, this is the first study on effects of nitrate, arginine, and ferrous on antibiotic recalcitrance of alginate-encapsulated clinical strains and at *mexY* expression level.

## Methods

### Bacterial strains and detection of biofilm formation by a colorimetric microtiter plate assay

A total of 10 *P. aeruginosa* mucoid strains isolated from cystic fibrosis patients’ sputum suffering from lung infection were received from hospital laboratories (see Additional file 1). To study biofilm formation ability of strains, a colorimetric microtiter plate assay was used. The strains were inoculated into R2A medium and incubated overnight at 37 °C. After incubation period, the cultures were diluted using the fresh R2A medium and the turbidity of bacterial suspensions was adjusted to 0.5 McFarland standard turbidity. 150 µl of each diluted culture was inoculated into a 96-well microtiter plate (the control well was the one containing uninoculated medium instead of bacterial suspension) and after 24 h incubation at 37 °C under static condition, bacterial suspensions were removed and wells (containing biofilms) were washed gently with PBS (three times). Then, 100 µl of methanol was added to wells and kept for 15 min to fix the biofilms; after removing methanol, 100 µl of crystal violet (1% v/w) was added to the wells, kept for 20 min, and then aspirated. Wells were washed with water, air-dried, and added with 150 µl of acetic acid (33% v/v). The optical density (OD) of each well was measured at 590 nm using a microtiter plate reader [18]. All assays were done in triplicate and the optical density (OD) of each well was measured at 590 nm. In order to interpret the results, a cut-off (ODc) was defined as average OD of control + (3 × standard deviation of control); and the OD of each well (strains) was compared with ODc. The strain was considered as a non-biofilm producing strain when OD ≤ ODc, a weak biofilm producing strain when ODc < OD ≤ 2.ODc, a moderate biofilm producing strain when 2 × ODc < OD ≤ 4 × ODc, and a strong biofilm producing isolate when 4 × ODc ≤ OD.

### Antimicrobial susceptibility testing

To determine MICs of amikacin, tobramycin, and ciprofloxacin which are used to treat *P. aeruginosa* infections in cystic fibrosis patients [19], the broth microdilution method was carried out according to the guidelines of the Clinical and Laboratory Standards Institute [20]. *P. aeruginosa* strain PAO1 and *Escherichia coli* strain ATCC 25922 were used as controls for MIC assays. For each antibiotic, a susceptible and a resistant or an intermediate strain were selected and subjected to further studies.

### Bacterial encapsulation into alginate beads

In order to encapsulate the bacterial strains into alginate beads, the modified method from Sønderholm et al*.* was used [10]. For this purpose, the strains were inoculated into R2A broth medium (24 h incubation at 37 °C) and then the suspension of each strain (OD_620nm_ 0.2) was added to the equal amount of alginate solution 3% (v/w) in R2A broth medium and were mixed well. After that, 250 µl of the resulting suspension was dropped slowly from an insulin syringe (a 1 ml insulin syringe with a 30-gauge needle attached) into wells of a 12-well microtiter plate containing cold calcium chloride solution (250 mM) to form beads with dimensions between 1–2 mm. and the beads were kept in calcium chloride solution for 1 h to harden. Then the solution was aspirated, the beads were washed with PBS, and 750 µl of R2A broth medium containing 50 mM calcium chloride was added to the beads. Beads were incubated at 37 °C for 24 h (100 rpm) to form bacterial biofilms.

### Confocal laser scanning microscopy for examination of microbial biofilms

In order to show the ability of the strains to produce aggregates in beads, the beads were stained with 0.1% (w/v) acridine orange for 2.5 min and then rinsed with PBS to remove excess stain. Beads were examined by confocal laser scanning microscope (Leica TCS SPE) with an excitation and emission filter of 488 nm and 505 nm, respectively [21].

### Determination of MBEC

To determine MBECs of ciprofloxacin, amikacin, and tobramycin against selected strains (strains 44-1, 73, 94-2, and 95-2), biofilms were formed into alginate beads by above mentioned method. Beads were washed with PBS and 750 µl of fresh R2A broth medium supplemented with 50 mM CaCl_2_ and different concentrations of antibiotics (serial two fold dilutions ranging from 5 to 5120 µg/mL [22]) were added to separate wells containing 250 µl beads. After incubation at 37 °C for 24 h (100 rpm), the culture medium was drained, beads were washed with PBS, and 750 µl of each citric acid and sodium carbonate solutions (final concentrations of 0.2 M) were added to beads and incubated for 20 min with constant shaking to dissolve them. The resulting suspension was pipetted several times and centrifuged for 3 min (5000 rpm). The pellet was washed and dissolved in 250 µl PBS and then serially diluted suspensions were inoculated on LB plates. After 24 h incubation at 37 °C, the colonies were counted and the lowest antibiotic concentration that inhibited the bacterial growth (no colonies on the agar plates) was considered as the MBEC [23].

### Effects of nitrate, arginine, and ferrous on antibiotic recalcitrance of bacterial strains

After determining MBECs, sub-MBEC concentration (antibiotics at concentrations one level below the determined MBEC) for each antibiotic was selected and used to study the effect of FeSO_4_ (0.5, 1, and 2 mM), arginine (0.2, 0.4, and 0.8% (w/v)), and KNO_3_ (50, 100, and 200 mM) on antibiotic resistance of encapsulated strains. After that, 250 µl of beads were prepared as noted above and placed in wells of a 12-well microtiter plate and 750 µl of R2A broth medium containing 50 mM calcium chloride was added to wells and beads were incubated at 37 °C (100 rpm) for 24 h. Then the culture medium was drained and beads were washed with PBS and freshly R2A medium containing 50 mM calcium chloride, each antibiotic with sub-MBEC concentration, and different concentrations of nitrate, arginine, or ferrous (as noted above) were added separately to the beads. Control groups of beads for each strain were included the following ones: one added with just freshly R2A medium containing 50 mM calcium chloride and the other one added with freshly R2A medium containing 50 mM calcium chloride and each antibiotic with the sub-MBEC concentration. After incubation at 37 °C (100 rpm) for 24 h, the medium was drained, beads were washed with PBS, and dissolved according to the method mentioned earlier. The resulting suspension was pipetted several times and centrifuged for 3 min (5000 rpm). After centrifugation, the supernatant was discarded and the pellet was washed with PBS, dissolved in 250 µL PBS, serially diluted, and inoculated on LB plates and incubated at 37 °C for 24 h. The colonies were counted and the number of colonies for each treated group were compared with the number of colonies from beads treated with just antibiotics.

### Real-time PCR analysis

Effect of ferrous on expression of the efflux pump gene, *mexY*, was studied on cystic fibrosis strains 95-2 and 44-1, and on the wild-type reference strain PAO1 as a control. Strains 44-1, 95-2 and also *P. aeruginosa* PAO1 were encapsulated into alginate beads according to the methods mentioned earlier; one group of beads was treated with only amikacin and another group was treated with amikacin and 0.5 mM FeSO_4_. The antibiotic concentration for strains 44-1, 95-2, and *P. aeruginosa* PAO1 was 320, 320, and 80 (µg/mL) which was sub-MBEC concentration of amikacin. Then, the beads were dissolved and suspensions were pipetted several times and then centrifuged for 3 min (5000 rpm); the supernatant was discarded, the cell pellet was washed with PBS, and then subjected immediately to RNA extraction procedure by using SinaPure-RNA kit (CinnaGen Co., Iran). The RNA solution was treated with DNase I (CinnaGen Co., Iran) to digest residual genomic DNA and then subjected to cDNA synthesis procedure by using cDNA synthesis kit (Yekta Tajhiz Azma Co., Iran).

The expression of *mexY* in control and treated groups was measured by real-time PCR with *rpsL* as the housekeeping gene [24]. Forward and reverse primers for PCR amplification of *mexY* (5′-GAAAGCTGGTCGATCCCG-3′ and 5′-TCAGGCCGACCTTGAAGTAG-3′) and *rpsL* (5′-AACGACCCTGCTTACGGTCT-3′ and 5′-ACGTCTGACCAACGGTTTCG-3′) were designed specifically for *P. aeruginosa* PAO1 (NCBI Genbank Accession number AE004091) and analyzed with Oligo Analyzer software version 1.0.3. Real-time PCR was performed using the StepOnePlus Real-Time PCR System (Applied Biosystems, USA) according to the manufacturer’s instructions. Triplicate reaction mixtures contained 2 µL cDNA were prepared using the RealQ Plus 2X master mix Green (Ampliqon, Denmark) in a total volume of 10 µL. The control contained no cDNA. Data were acquired at 72 °C and the melting curves were observed to verify primer specificity and real-time PCR data were analyzed by 2^−ΔΔC^_t_ method, which is used to determine the fold difference in gene expression by using the following equation [25]:

$$\Delta {\text{C}}_{{\text{t Treated sample}}} = {\text{ C}}_{{{\text{t Gene of interest }}(mexY)}} - {\text{ C}}_{{{\text{t Housekeeping gene }}(rpsL)}}$$

$$\Delta {\text{C}}_{{\text{t Untreated sample}}} = {\text{ C}}_{{{\text{t Gene of interest }}(mexY)}} - {\text{ C}}_{{{\text{t Housekeeping gene }}(rpsL)}}$$

$$\Delta \Delta {\text{C}}_{{\text{t}}} = \, \Delta {\text{C}}_{{\text{t Treated sample}}} - \, \Delta {\text{C}}_{{\text{t Untreated sample}}}$$

### Statistical analyses

GraphPad Prism 9 (GraphPad Software Inc., USA) was used to analyze the data. Differences between treated groups and controls were determined by using one-way ANOVA and *t* test. P values < 0.05 were considered as statistically significant.

## Results

### MIC and MBEC determination and biofilm formation by the strains

Determination of MICs of amikacin, tobramycin, and ciprofloxacin as well as the ability to form biofilm were assessed for the 10 cystic fibrosis and PAO1 strains; the results are shown in Table 1. Strain 44-1 was sensitive to all the three antibiotics and had a weak biofilm formation ability. Strains 94-2 and 73 were resistant to ciprofloxacin and tobramycin, respectively; neither strain was resistant to amikacin.

**Table 1:** MIC and MBEC values of antibiotics and ability of the strains to form biofilm

Strain name		Amikacin			Tobramycin			Ciprofloxacin			Biofilm formation
		S/I/R^b^	MIC^c^	MBEC^c^	S/I/R	MIC	MBEC	S/I/R	MIC	MBEC	(N/W/M/S)^d^
Strains isolated from CF^a^ patients	98–4	S	8	–	S	0.5	–	S	0.5	–	M
	30–1	S	16	–	S	2	–	S	1	–	W
	44–1	S	8	640	S	1	640	S	0.5	160	W
	95–1	S	4	–	S	0.25	–	I	2	–	M
	95–2	I	32	640	S	0.25	–	I	2	–	M
	96–3	S	8	–	S	0.25	–	I	2	–	M
	84–3	S	8	–	S	1	–	R	8	–	S
	94–2	I	32	–	S	1	–	R	8	160	S
	73	I	32	–	R	32	640	R	8	–	W
	98–3	I	32	–	S	1	–	S	0.5	–	S
Reference strains	PAO1	S	2	160	S	0.5	10	S	0.125	20	M

^a^CF stands for cystic fibrosis

^b^S stands for sensitive, I stands for intermediate-resistant, and R stands for resistant

^c^The values are given as (µg/mL)

^d^N stands for non-biofilm producing, W stands for weak biofilm producing, M stands for moderate biofilm producing, S stands for strong biofilm producing

**Fig. 1:**
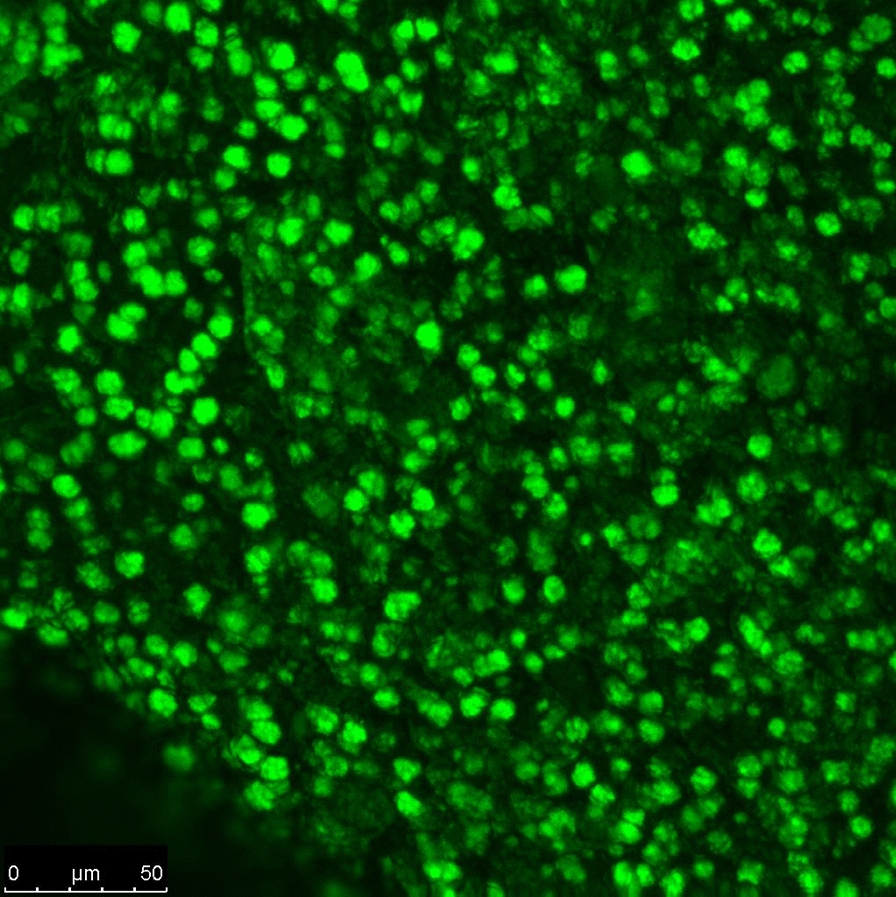
Confocal laser scanning microscope image of strain 44-1 aggregates

As mentioned earlier, one susceptible and one resistant or an intermediate-resistant strain (including strains 44-1, 73, 94-2, and 95-2) were selected for each antibiotic and subjected to MBEC determination and study the effect of supplementation. Evaluation of biofilms by confocal microscopic analysis showed that the diameter of aggregates were around 10 µm, which was similar to other strain aggregates (data not shown). The 3-dimension visualization of strain 44–1 aggregates is shown in Fig. 1.

**Fig. 2:**
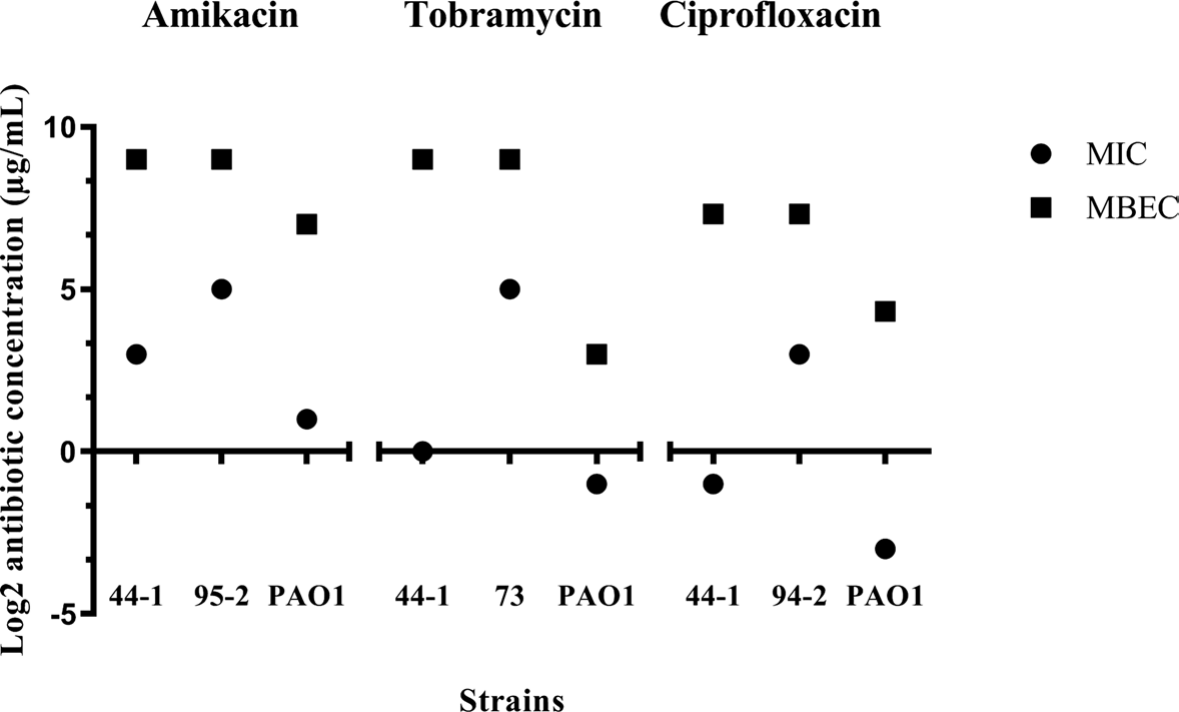
Comparison between MIC and MBEC values of antibiotics for selected strains (strains 44-1, 73, 94-2, and 95-2)

According to the Fig. 2, increased biofilm resistance (MBEC > MIC) was observed for selected strains towards antimicrobial agents. MBEC values were 80 and 20 times greater than MIC values for strains 44-1 and 95-2, respectively. The difference between these values for tobramycin and ciprofloxacin was 640 and 20 times for strains 44-1 and 73, and 320 and 20 times for strains 44-1 and 94-2, respectively (Table 1).

### Effects of nitrate, arginine or ferrous supplementation

From 10 clinical strains, four strains were selected to study the effect of supplements in combination with antibiotics: one susceptible to all antibiotics (44-1), two resistant to tobramycin and ciprofloxacin (73 and 94-2), and one exhibiting an intermediate resistance to amikacin (95-2, as no amikacin resistance was found among the 10 strains). For each antibiotic, the susceptible and control strains (44-1 and PAO1) were compared with a resistant one.

**Fig. 3:**
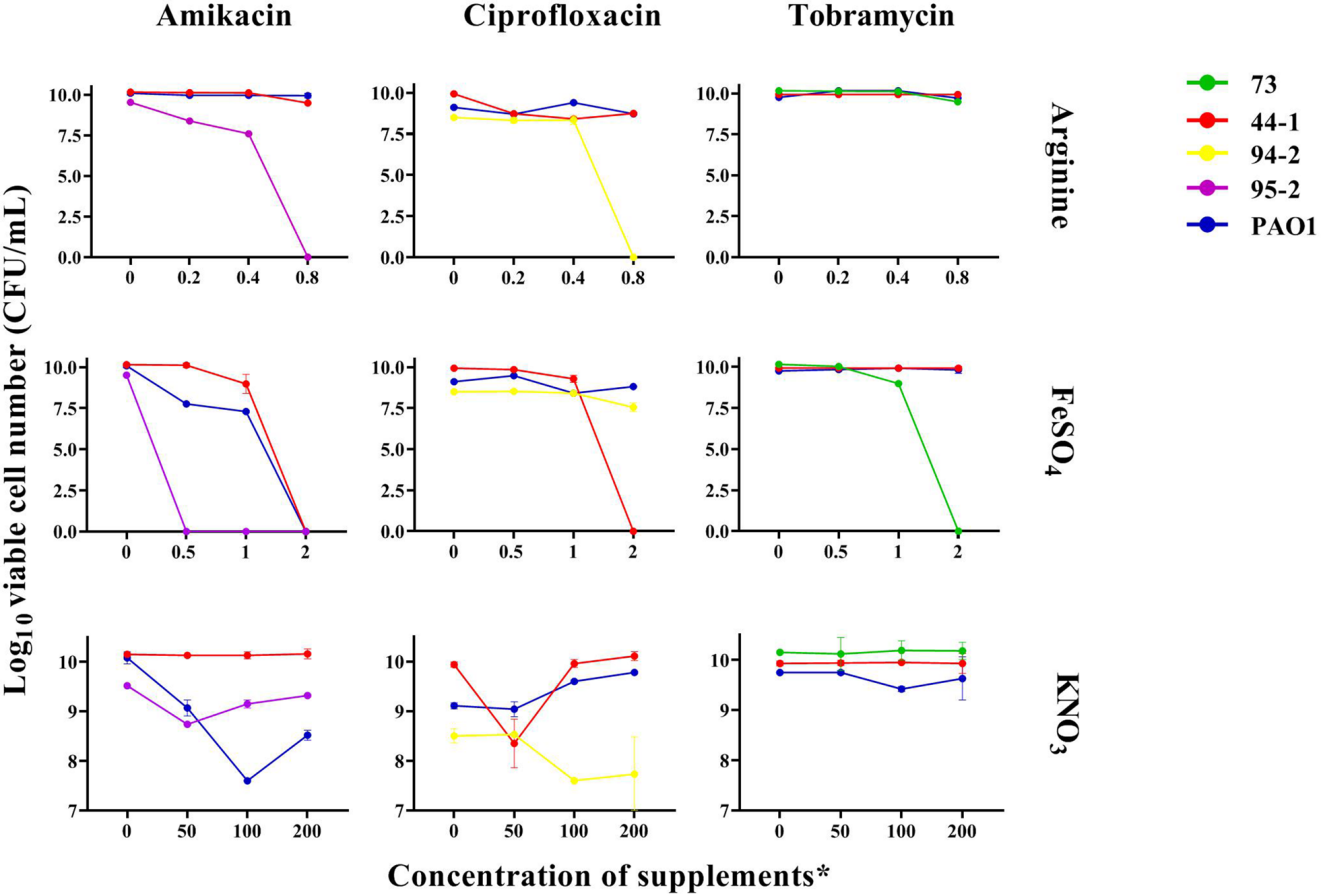
Effect of nitrate, arginine, and ferrous in combination with antibiotics on antibiotic resistance. *Concentrations are based on mM for FeSO_4_ supplementations and % (v/w) for arginine and KNO_3_ supplementations

Results of the experiments on investigation of the effects of nitrate, arginine, and ferrous supplementation in combination with antibiotics on reducing the number of cells in alginate beads for evaluated strains are shown in Fig. 3. According to the results, these supplements had a dose-related effect on decreasing the number of viable cells and maximal effect was noted with ferrous, as the reduction of cells for most strains was statistically significant in the presence of ferrous and all the three antibiotics, followed by arginine and nitrate. One mM FeSO_4_ in combination with amikacin eradicated all the strain 95-2 cells (P value < 0.0001) and resulted in a 1.2 (P value < 0.0001) and 2.8 (P value < 0.0001) log reduction in strains 44-1 and *P. aeruginosa* PAO1, respectively. The significant reduction of CFUs (colony-forming units) at 1 mM FeSO_4_ was also observed in the presence of tobramycin for strain 73 which showed a 1.2 (P value < 0.0001) log reduction in viable cells and in the presence of ciprofloxacin for strains 44-1 and *P. aeruginosa* PAO1, which showed a 0.6 (P value = 0.0014) and 0.7 (P value < 0.0001) log reduction, respectively. The efficacy of killing by antibiotics increased by increasing FeSO_4_ concentration to 2 mM. At this concentration, amikacin killed all cells of the tested strains and ciprofloxacin was able to kill all cells of strain 44–1 and give a 0.9 (P value = 0.0036) and 0.3 (P value = 0.0001) log reduction in the number of viable cells of strains 94-2 and *P. aeruginosa* PAO1, respectively. The reduction of CFUs resulting from treatment with 0.2% (w/v) arginine was significantly different with control groups for amikacin-treated strain 95-2 (P value < 0.0001) and ciprofloxacin-treated strains 44-1 and *P. aeruginosa* PAO1 (P value < 0.0001 for both strains). 0.4% (w/v) arginine gave a 1.9 (P value < 0.0001) and 1.5 (P value < 0.0001) log reduction in CFUs of strain 95-2 in the presence of amikacin and strain 44-1 in the presence of ciprofloxacin, respectively. 0.8% (w/v) arginine was capable of eradicating completely all viable cells of amikacin-treated strain 95-2 (P value < 0.0001) and ciprofloxacin-treated strain 94-2 (P value < 0.0001). At this concentration, there were statistically significant viable cell reduction in strain 44-1 (in the presence of amikacin), 73 (in the presence of tobramycin), and 44-1 and *P. aeruginosa* PAO1 (in the presence of ciprofloxacin).

The least effective supplement in killing bacteria in combination with antibiotics was nitrate, as the reduction of cells was less significant in comparison to other supplements. Among the three tested concentrations of KNO_3_, cell treatments with antibiotics and 100 mM KNO_3_ achieved the most reduction in viable cell numbers. At this concentration, the reduction of viable cells was statistically significant for strains 95-2 and *P. aeruginosa* PAO1 in combination with amikacin and strain 94-2 in combination with ciprofloxacin and the log reduction of CFUs was 0.5 (P value < 0.0001), 2.5 (P value < 0.0001), and 0.9 (P value = 0.0058), respectively.

Among all strains, strain 95-2 was the most susceptible one to supplementations and the reduction of cells for all concentrations of the three supplements was statistically significant and all concentrations of ferrous were helpful to eradicate the cells completely. The least susceptible strain was tobramycin-treated strain 44-1, as none of the supplementations was able to statistically significant reduce the CFU (see Additional file 2).

### Determination of *mexY* expression by real-time PCR

After interpreting the data, amikacin which its combination with ferrous showed the most significant eradication of the tested strains (95-2, 44-1, and PAO1) was selected and further studies on the effect of ferrous on expression of the efflux pump gene, *mexY*, were carried on those strains.

**Fig. 4:**
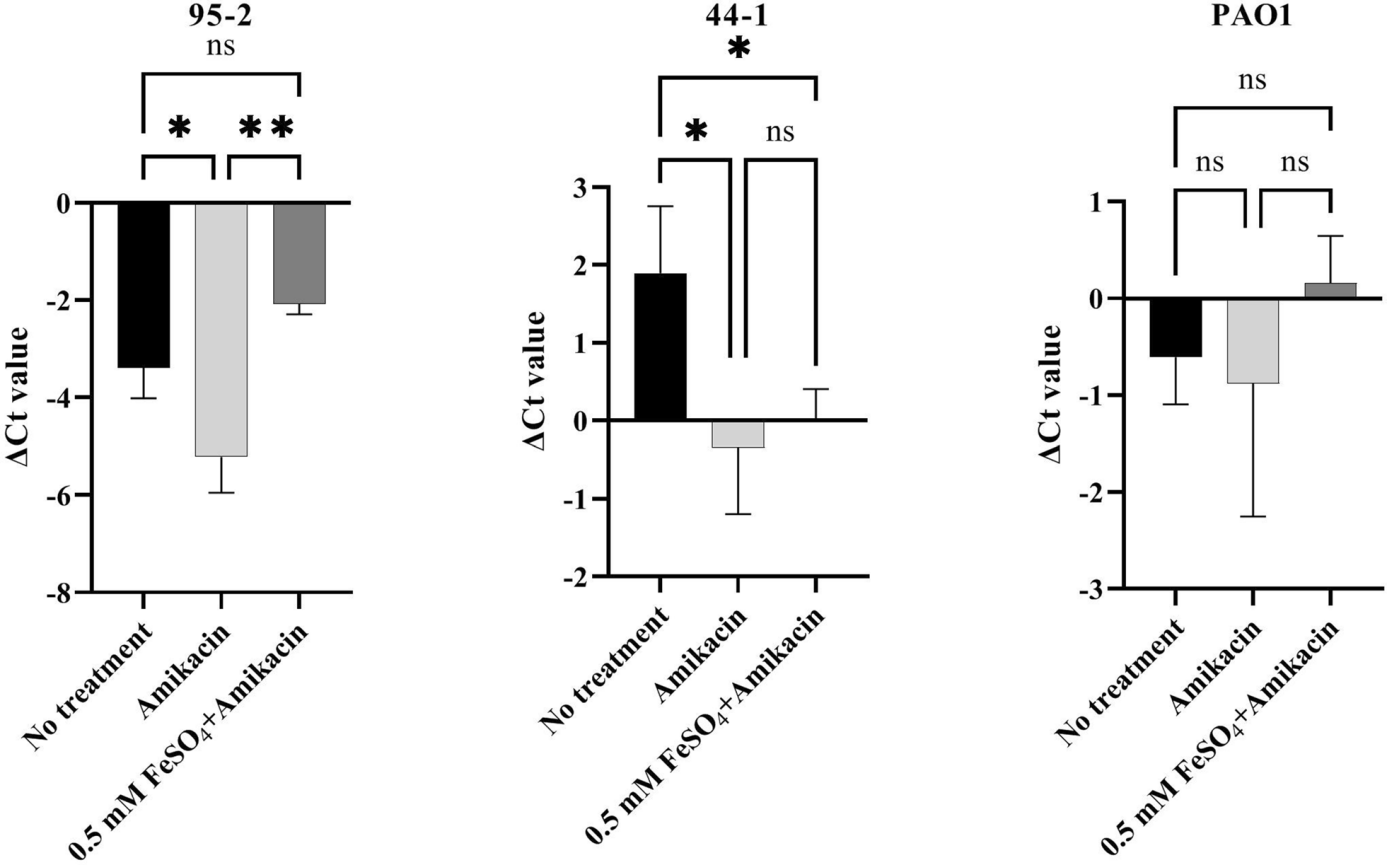
Determination of *mexY* expression in strains 95-2, 44-1, and PAO1 by Real-time PCR. Expression was normalized to *rpsL* gene. Data represent the mean value of three biological replicates and error bars indicate standard deviation. One-way ANOVA test: *P < 0.05; **P < 0.01

**Fig. 5:**
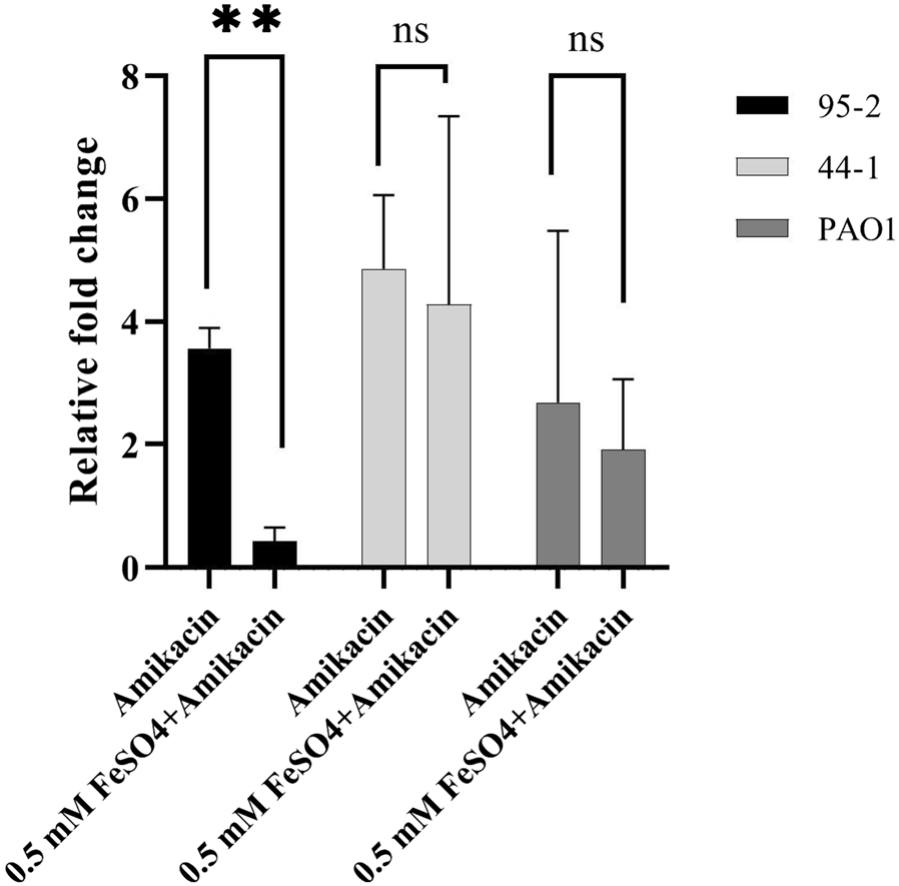
Relative fold changes determined by Real-time PCR analysis of *mexY* gene. Relative fold changes show expression level of *mexY* gene in treated groups (only amikacin and amikacin + 0.5 mM FeSO_4_) relative to the untreated (control) group for each strain. Data represent the mean value of three biological replicates and error bars indicate standard deviation. T test: **P < 0.01

The expression of *mexY* gene was determined by using real-time PCR in the following three groups for 95-2, 44-1, and PAO1 strains: untreated, treated with amikacin, and treated with amikacin and 0.5 mM FeSO_4_. Data analysis showed increase in the expression of *mexY* in the alginate-encapsulated strains treated with amikacin in comparison with controls*.* The increase was 3.6-fold for strain 95-2 and for 44-1 and PAO1 strains the value was 4.8 and 2.6, respectively. According to Fig. 4, treating strain 95-2 with ferrous and amikacin showed a significant decrease in expression of *mexY* in comparison with control and the group that was treated with just amikacin. The relative expression level of *mexY* in strain PAO1 was 4.7 times more than strain 95-2 when the strains were treated with the combination of ferrous and amikacin. The relative fold changes compared to the control (untreated group) for each strain is shown in Fig. 5.

## Discussion

Biofilm related infections are resistant to antibiotics and difficult to treat. Many studies have been focused on developing biofilm models with closest characteristics to natural biofilms to find appropriate treatments to eradicate biofilms. It has been shown that alginate-encapsulated *P. aeruginosa* is a good in vitro model that mimics characteristics of *P. aeruginosa* aggregates in lung and wound infections [10]. In the present study, effects of nitrate, arginine, and ferrous were determined on antibiotic recalcitrance of *P. aeruginosa* strains in alginate beads in the presence of amikacin, tobramycin, and ciprofloxacin. The diameter of aggregates in alginate beads were mostly around 10 µm which was similar to the diameter of aggregates of *P. aeruginosa* isolated from chronic infections (5–50 µm) [26]. Totally, the results were promising in this study, but further experiments need to be done (especially on planktonic cells) to elaborate on whether the observed effects were general or biofilm-specific. Among the strains, only strain 44-1 did not show any response towards most of the antibiotic-supplement combinations. As the strain 44-1 was antibiotic susceptible (according to the MICs) with a high MBEC, this very high biofilm-induced tolerance might explain why most of the antibiotic-supplement combinations were not effective.

Biofilms limit diffusion of oxygen into their matrix resulting in hypoxic or anoxic microenvironment in the biofilms. This situation restricts bacterial growth and makes them become metabolically inactive or dormant and consequently, more resistant to antibiotics [27]. *P. aeruginosa* is able to use other electron acceptors like nitrate and arginine, in hypoxic or anoxic conditions. When biofilm or bacterial aggregates are forming in the laboratory in the presence of nitrate, *P. aeruginosa* grows deeper in the biofilm as the oxygen limitation is not a problem anymore and the result is more antibiotic resistance [10, 28]. On the other hand, when an already-formed biofilm is treated with nitrate, it activates metabolically inactive bacterial cells which makes them more susceptible to antibiotics [29]. In the present study, it was shown that different concentrations of nitrate can increase or decrease antibiotic susceptibility of *P. aeruginosa* strains when aggregates of this bacterium were treated with a combination of nitrate and an antibiotic. The concentration of nitrate could be as high as 600 µM in cystic fibrosis patient’s sputum [30] which is lower than nitrate concentrations used in this study. According to the results, the effect of nitrate on antibiotic susceptibility of strains was dependent on the type of antibiotics and nitrate concentration. As for the type of antibiotics, nitrate was less effective when it was used in combination with tobramycin which was similar to previous reports [28, 31]. The dependence of nitrate efficacy on the type of antibiotics was also found by Borriello and co-workers [28]. With respect to nitrate concentration, strain 95-2 which had intermediate resistance to amikacin, was more susceptible to amikacin when it was treated with 50 mM nitrate, but it did not become more susceptible when the nitrate concentration was increased. The same pattern of not increasing antibiotic susceptibility by increasing nitrate concentration was observed for strain 44-1 (which was ciprofloxacin-susceptible), strain 94-2 (which was ciprofloxacin-resistant), and also for strain PAO1 when it was treated with all the three antibiotics. The decrease in antibiotic susceptibility of *P. aeruginosa* PAO1 in the presence of nitrate has been reported previously [28]. The increased susceptibility to antibiotics in the presence of nitrate is due to the activation of dormant cells and the antibiotic effect on these metabolically active cells. In addition, the biofilm response to antibiotics in the presence of nitrate has been shown to be related to the age of the biofilm [29].

As it was mentioned earlier, in the absence of oxygen and presence of arginine, *P. aeruginosa* uses arginine as a final electron acceptor to stay metabolically active [32]. According to the previous publications, arginine concentration in cystic fibrosis patient’s sputum is 11.7 µM; and increased activity of arginase in these patients results in decreasing the bioavailability of arginine in their airways [33]. The arginine concentration in the present study experiments was 12, 24, and 48 mM (0.2, 0.4, and 0.8% (v/w), respectively) which was higher than the amount of this amino acid in cystic fibrosis patient’s sputum. Studies on the effect of different concentrations of arginine on antibiotic susceptibility of *P. aeruginosa* aggregates in alginate beads showed that arginine increased amikacin susceptibility of strain 95-2 in a way that 0.8% (v/w) arginine eradicated all the bacteria. Also, 0.8% (v/w) arginine killed all the strain 94-2 cells when they were treated with ciprofloxacin and arginine. As it was said about nitrate, arginine effect is also dependent on its concentration. For example, PAO1 showed more tolerance against tobramycin when it was treated with tobramycin and 0.2 and 0.4% (v/w) arginine, but the tolerance decreased in the presence of 0.8% (v/w) arginine. For all the bacteria and antibiotics tested in this study, 0.8% (v/w) arginine showed the best result regarding enhancement of antibiotic effects. Similarly, Borriello et al. showed that arginine enhanced eradication of *P. aeruginosa* in biofilms when they were treated with arginine and ciprofloxacin or tobramycin [29].

Our study showed that ferrous was the most effective supplement to enhance antibiotic effects in comparison with nitrate and arginine. Two mM ferrous was able to kill all alginate-encapsulated *P. aeruginosa* strains 95-2, 44-1 (amikacin sensitive), 73, 44-1 (ciprofloxacin sensitive), and strain PAO1 (treated with amikacin). Yeom and coworkers showed that iron is an important factor for antibiotic actions [34]. In another investigation, iron salts including ferrous sulfate had anti-biofilm activities. The authors stated that there is an optimum range of iron concentration for *P. aeruginosa* to form biofilms and above or below that range, the bacterium is not able to form biofilm and it can only have planktonic lifestyle [17]. In human air ways, iron is mostly found in the form of bound to transferrin, lactoferrin, and ferritin which are iron binding proteins and the concentration of free iron is completely low [35], about 8 µM in sputum of cystic fibrosis patients [36]. *P. aeruginosa* needs iron-chelating compounds called siderophores to get iron from transferrin, lactoferrin, and ferritin and transfer iron into its cell [35]. Moreover, *P. aeruginosa* is able to take up free ferrous by using its Feo system [37].

We also evaluated the effects of amikacin and the combination of amikacin and ferrous on expression of *mexY* efflux pump because strain 95-2 was the only strain that was eradicated completely when it was treated with the antibiotic (amikacin) and all the three concentration of the tested supplement (ferrous). We wanted to confirm the hypothesis that down-regulation of *mexY* was the reason for complete eradication of strain 95-2 in the presence of ferrous and amikacin. Efflux systems pump most antibiotics out from the bacterial cells, but they act selectively and are effective on special groups of antibiotics. MexXY-OprM is an efflux system responsible for *P. aeruginosa* resistance to aminoglycoside antibiotics like amikacin. It has been proven that treating of *P. aeruginosa* with some antibiotics like amikacin leads to overexpression of efflux pump genes in this bacterium [38, 39]. Sobel et al. have reported that MexXY-OprM is expressed in some *P. aeruginosa* strains even when they are not treated with antibiotics and in some strains it is not overexpressed in the presence of antibiotics [40]. In the present study, it was observed that *mexY* was significantly overexpressed when strains 95-2 and 44-1 were treated with amikacin. It means the expression of *mexY* was increased in the presence of amikacin; however, *mexY* was already expressed in the strains in the absence of antibiotics and its expression level in these strains was different. Also, the results showed that the combination of amikacin and ferrous was significantly effective on reducing the expression of this gene in an amikacin-resistant strain 95-2, which is a promising result. As iron regulates the expression of some genes in *P. aeruginosa* [16], it can be hypothesize that high concentration of iron down-regulates the expression of *mexY* which means more antibiotic molecules remain in the cell and kill the bacterium.

## Conclusions

The present study, represents the effects of nitrate, arginine, and ferrous supplementation on reducing the number of viable cells and *mexY* expression in clinical *P. aeruginosa* strains in a biofilm model, for the first time. As the findings are promising, addition of ferrous or arginine supplements to the drug regimen of animal models suffering from *P. aeruginosa* infections can be considered in in vivo experiments in the future. Moreover, it is possible that the expression of other virulence factors get affected by these supplements in this biofilm model, also the combination of supplements could be more effective on eradication of bacterial cells and the hypotheses need to be evaluated in further studies.

## Supplementary Information

**Additional file 1.** Information about the clinical strains. The data represent results of oxidase and antibiogram tests on strains, shows if the strains are mucoid and produce pigments, and some information about the patients that the strains have been isolated from.

**Additional file 2. **Effect of nitrate, arginine, and ferrous in combination with antibiotics on antibiotic resistance. The data represent results of studying effect of different concentrations of nitrate, arginine, and ferrous on antibiotic resistance of selected strains in the presence of amikacin, tobramycin, and ciprofloxacin.

## Data Availability

Not applicable.

## Abbreviations

MIC: Minimum inhibitory concentration
MBEC: Minimal biofilm eradication concentration
RND: Resistance-nodulation-division
CFU: Colony-forming units
Fig: Figure
