# Supplementary material for: Determining effects of nitrate, arginine, and ferrous on antibiotic recalcitrance of clinical strains of Pseudomonas aeruginosa in biofilm-inspired alginate encapsulates
Source: Ann Clin Microbiol Antimicrob. 2023 Jul 20;22:61. doi: 10.1186/s12941-023-00613-y (PMC10360276; doi:10.1186/s12941-023-00613-y)
Supplement: Supplementary file 1 — Additional file 1. Information about the clinical strains. The data represent results of oxidase and antibiogram tests on strains, shows if the strains are mucoid and produce pigments, and some information about the patients that the strains have been isolated from. [file 12941_2023_613_MOESM1_ESM.pdf]

Information about the clinical strains.

| Strain | Oxidase | Mucoid | Pigmentation Phenotype* | Antibiogram results† (S/I/R) ‡ | Chronic infection | Patient's age (year) |
|---|---|---|---|---|---|---|
| 98-4 | + | + | + | IMI, (S); CAZ, (S); MEM, (S); CPM, (R); ATM, (S); GM, (R); LEV (R) | + | 26 |
| 30-1 | + | + | + | IMI, (S); CAZ, (S); MEM, (S); CPM, (I); ATM, (S); GM, (R); LEV (S) | + | 13 |
| 44-1 | + | + | + | IMI, (R); CAZ, (S); MEM, (S); CPM, (R); ATM, (S); GM, (R); LEV (S) | + | 22 |
| 95-1 | + | + | + | IMI, (S); CAZ, (S); MEM, (S); CPM, (I); ATM, (S); GM, (R); LEV (R) | + | 20 |
| 95-2 | + | + | + | IMI, (S); CAZ, (S); MEM, (S); CPM, (R); ATM, (S); GM, (R); LEV (R) | + | 20 |
| 96-3 | + | + | + | IMI, (S); CAZ, (S); MEM, (S); CPM, (R); ATM, (S); GM, (R); LEV (R) | + | 15 |
| 84-3 | + | + | + | IMI, (S); CAZ, (S); MEM, (S); CPM, (R); ATM, (S); GM, (R); LEV (R) | + | 25 |
| 94-2 | + | + | + | IMI (S), CAZ (S), MEM (S), CPM (I), ATM (S), GM (S), LEV (R) | + | 31 |
| 73 | + | + | + | IMI, (S); CAZ, (S); AZT, (R); GM, (R); CTR, (S) | + | - |
| 98-3 | + | + | + | IMI, (S); CAZ, (S); MEM, (S); CPM, (S); ATM, (S); GM, (S); LEV, (I) | + | 17 |

* Pigmentation phenotype refers to any pigment that was produced by the strains, not a specific pigment; †Antibiotic names are given as abbreviations: IMI, Imipenem; CAZ, Ceftazidime; MEM, Meropenem; CPM, Cefepime; ATM, Aztreonam; GM, Gentamicin; LEV, Levofloxacin; AZT, Aztreonam; CTR, Ceftriaxone; ‡S stands for sensitive, I stands for and intermediate-resistant, and R stands for resistant
