# Supplementary material for: Determining effects of nitrate, arginine, and ferrous on antibiotic recalcitrance of clinical strains of Pseudomonas aeruginosa in biofilm-inspired alginate encapsulates
Source: Ann Clin Microbiol Antimicrob. 2023 Jul 20;22:61. doi: 10.1186/s12941-023-00613-y (PMC10360276; doi:10.1186/s12941-023-00613-y)
Supplement: Supplementary file 2 — Additional file 2. Effect of nitrate, arginine, and ferrous in combination with antibiotics on antibiotic resistance. The data represent results of studying effect of different concentrations of nitrate, arginine, and ferrous on antibiotic resistance of selected strains in the presence of amikacin, tobramycin, and ciprofloxacin. [file 12941_2023_613_MOESM2_ESM.pdf]

Effect of nitrate, arginine, and ferrous in combination with antibiotics on antibiotic resistance.

| Treatment / Strain | Amikacin $Log_{10}$ viable cell number (CFU/mL) | | | Tobramycin $Log_{10}$ viable cell number (CFU/mL) | | | Ciprofloxacin $Log_{10}$ viable cell number (CFU/mL) | | |
|---|---|---|---|---|---|---|---|---|---|
| | **95-2** | **44-1** | **PAO1** | **73** | **44-1** | **PAO1** | **94-2** | **44-1** | **PAO1** |
| Control | 9.52±0.02 | 10.15±0.03 | 10.08±0.12 | 10.15±0.03 | 9.93±0.05 | 9.75±0.01 | 8.5±0.14 | 9.94±0.05 | 9.11±0.06 |
| 0.2% (w/v) Arg†+AB | 8.38±0.06**** | 10.12±0.07 | 9.95±0.08 | 10.13±0.13 | 9.93±0.03 | 10.15±0.01 | 8.33±0.16 | 8.73±0.03**** | 8.7±0.03**** |
| 0.4% (w/v) Arg+AB | 7.59±0.02**** | 10.11±0.01 | 9.95±0.08 | 10.11±0.1 | 9.93±0.04 | 10.15±0.06 | 8.34±0.25 | 8.41±0.07**** | 9.4±0.07*** |
| 0.8% (w/v) Arg+AB | 0±0**** | 9.48±0.02** | 9.94±0.14 | 9.48±0.02*** | 9.93±0.04 | 9.71±0.06 | 0±0**** | 8.75±0.06**** | 8.72±0.04**** |
| 50 mM $KNO_3$+AB | 8.74±0.01**** | 10.13±0.01 | 9.07±0.16**** | 10.12±0.33 | 9.94±0.05 | 9.75±0.04 | 8.53±0.02 | 8.35±0.49**** | 9.04±0.15 |
| 100 mM $KNO_3$+AB | 9.15±0.08**** | 10.13±0.07 | 7.6±0.01**** | 10.19±0.19 | 9.95±0.03 | 9.42±0.05 | 7.6±0.02** | 9.96±0.08 | 9.6±0.03**** |
| 200 mM $KNO_3$+AB | 9.32±0.02**** | 10.16±0.1 | 8.52±0.1**** | 10.18±0.17 | 9.93±0.2 | 9.63±0.43 | 7.73±0.75* | 10.11±0.09 | 9.78±0.02**** |
| 0.5 mM $FeSO_4$+AB | 0±0**** | 10.12±0.13 | 7.77±0.06**** | 10.02±0.04 | 9.93±0.05 | 9.84±0.1 | 8.53±0.06 | 9.85±0.08 | 9.48±0.06**** |
| 1 mM $FeSO_4$+AB | 0±0**** | 8.98±0.58**** | 7.3±0.06**** | 8.98±0.05**** | 9.92±0.04 | 9.92±0.07 | 8.41±0.07 | 9.29±0.22** | 8.41±0**** |
| 2 mM $FeSO_4$+AB | 0±0**** | 0±0**** | 0±0**** | 0±0**** | 9.92±0.05 | 9.81±0.21 | 7.56±0.27** | 0±0**** | 8.82±0.02*** |

‡ Arg stands for arginine. Asterisks show the significance of decreased or increased resistance of treated strains with supplements in comparison with controls. One-way ANOVA test: *, P value < 0.05; **, P value < 0.01; ***, P value < 0.001; ****, P value < 0.0001.
